# Metreleptin therapy in *LMNA*-linked lipodystrophies

**DOI:** 10.1186/1750-1172-10-S2-O27

**Published:** 2015-11-11

**Authors:** Camille Vatier, Corinne Vigouroux

**Affiliations:** 1 INSERM UMR_S938, Centre de Recherche Saint-Antoine, Sorbonne Universités UPMC Univ Paris 06, ICAN, Institute of Cardiometabolism and Nutrition, Paris, France

## 

Lipodystrophic syndromes are rare diseases of acquired or genetic origin, associating a decreased amount of fat (with an altered distribution of body fat in partial forms) and the metabolic alterations usually observed in obesity, i.e. insulin resistance leading to diabetes, hypertriglyceridemia with the risk of acute pancreatitis, fatty liver with risk of cirrhosis, and precocious atherosclerosis. Mutations in more than 15 genes, including *LMNA*, have been shown to be responsible of monogenic forms of lipodystrophies. The decreased capacity of adipocytes to store excess energy as lipids and to perform physiological endocrine functions, is considered as the main pathophysiological determinant of lipodystrophies. The low circulating levels of leptin lead to an increased appetite and participate in the ectopic storage of lipids in the muscle and liver, which aggravates the metabolic alterations. Replacement leptin therapy was shown to strikingly improve insulin resistance, dyslipidemia and liver steatosis in patients with generalized form of lipoatrophy, associated with very low endogenous secretion of leptin[1]. Recombinant leptin (metreleptin), administrated in one daily subcutaneous injection, is well-tolerated, and, although it did not improve lipoatrophy itself, demonstrated metabolic benefits in 55 lipodystrophic patients during a 3-year therapy[2]. Regarding laminopathies, two studies evaluated metreleptin therapy in 6 then 24 patients with the Dunnigan-type familial partial lipodystrophy[3,4]. Although metreleptin was efficient in decreasing circulating triglycerides and liver steatosis, the effects on glucose homeostasis did not reach statistical significance. Metreleptin, which is the first specific therapy for lipodystrophies, was approved in 2014 by the FDA for generalized forms, and is available in selected European centers through compassionate programs. Further studies are needed to clarify the therapeutic indications of metreleptin in partial lipodystrophies including laminopathies.
